# Optimal Oversizing Index Depending on Valve Type and Leakage-Proof Function for Preventing Paravalvular Leakage after Transcatheter Aortic Valve Implantation

**DOI:** 10.3390/jcm9123936

**Published:** 2020-12-04

**Authors:** You-Jeong Ki, Jeehoon Kang, Hak Seung Lee, Mineok Chang, Jung-Kyu Han, Han-Mo Yang, Kyung Woo Park, Hyun-Jae Kang, Bon-Kwon Koo, Hyo-Soo Kim

**Affiliations:** Cardiovascular Center, Seoul National University Hospital, Seoul 03080, Korea; drkiyou@gmail.com (Y.-J.K.); medikang@gmail.com (J.K.); cardiolee@gmail.com (H.S.L.); oklizard81@gmail.com (M.C.); hpcrates@gmail.com (J.-K.H.); hanname@gmail.com (H.-M.Y.); kwparkmd@snu.ac.kr (K.W.P.); nowkang@snu.ac.kr (H.-J.K.); bkkoo@snu.ac.kr (B.-K.K.)

**Keywords:** transcatheter aortic valve implantation, oversizing index, leakage-proof function, paravalvular leakage

## Abstract

Paravalvular leak (PVL) is an important complication of transcatheter aortic valve implantation (TAVI) and is associated with poor prognosis. We aimed to identify the risk factors for PVL after TAVI including patient (calcium amount or location), device (leakage-proof or not), and procedural (oversizing index (OI)) factors. The primary outcome was mild or greater PVL at 1-month follow-up echocardiography. Overall, 238 patients who underwent TAVI using eight types of valves (Edwards Sapien, Sapien XT, Sapien 3, CoreValve, Evolut R, Evolut PRO, Lotus, and Lotus Edge) were included. The incidence of significant PVL (≥mild PVL) was 24.4%. Although patient factors (calcification of valve) were not predictors of PVL, valve without leakage-proof function (Edwards Sapien, Sapien XT, and CoreValve) was a significant predictor of PVL (adjusted odds ratio, 3.194, 95% CI, 1.620–6.299). Furthermore, OI has a significant protective role against PVL (PVL increased by 45% when OI decreased by 5%). The best cutoff value of OI to predict the absence of PVL was ≥17.6% for the Evolut system and ≥10.2% for the Sapien system. The predictors of PVL after TAVI included factors from the device (valve without leakage-proof function) and procedure (under-sizing). In patients with a high risk of PVL, the procedure should be optimized using valves with leakage-proof function and adequate OI.

## 1. Introduction

Transcatheter aortic valve implantation (TAVI) has emerged as the new standard treatment of severe aortic stenosis (AS) in patients at intermediate and high surgical risk [1,2,3]. TAVI is non-inferior to surgery in terms of composite death or stroke in severe AS with low surgical risk patients [4]. Even in another study conducted in low-risk patients, TAVI performed with balloon-expandable valve was superior to surgery [5].

However, the risk for paravalvular leakage (PVL) is one of the main limitations of TAVI procedure, which is associated with increased mortality during follow-up [1,6]. Although it was generally believed that moderate or severe PVL impact long term clinical outcomes, the recently published studies showed that even mild PVL was associated with mortality. As the indications of TAVI were expanded, mild PVL may also be important for long-term clinical outcomes [1,6,7]. The factors linked to PVL and mortality were increased left ventricular (LV) workload, infective endocarditis, and hemolysis [8,9,10].

The well-known PVL associated factors include calcification of aortic valve (AV) or device landing zone [11], newer/older version of the valve, and periprocedural factors [12,13]. The patient factor is calcification in the annulus and the left ventricular outflow tract (LVOT), which creates the gap between the TAVI valve and the patient’s tissues, where PVL occurs. Device factors have roles in overcoming the AV calcification. Through the development of a unique mechanism of sealing the fabric around the TAVI valve, the incidence of PVL was reduced in the recent TAVI era [14]. Finally, procedural factors are important because physicians can easily determine and adjust for the best result. Particularly, the oversizing index (OI) is used to reduce the PVL after TAVI [12]. The OI indicates the degree to which the valve size is greater compared to the annular size. Although a higher OI could reduce PVL, the impact of OI on PVL is not consistent and the optimal OI has not yet been established. This study aimed to assess the predictors of PVL from the patient, device, and procedural factors. We propose the best procedural protocol to reduce PVL by providing optimal OI for different types of valves.

## 2. Methods

### 2.1. Study Population

From July 2011 and February 2020, 287 consecutive patients underwent TAVI procedure at Seoul National University Hospital for symptomatic severe AS. Patient data were prospectively collected in the Seoul National University Hospital TAVI registry and retrospectively analyzed. All patients underwent pre-procedural multidetector computed tomography (MDCT). Patients who underwent 1-month follow-up echocardiography were enrolled in this study. This study complied with the provisions of the Declaration of Helsinki. The study protocol was approved by the institutional review board of Seoul National University Hospital. All patients provided written informed consent.

### 2.2. Definitions and Outcomes

The valves used in this trial included Edwards Sapien, Sapien XT, Sapien 3, CoreValve, Evolut R, Evolut PRO, Lotus, and Lotus Edge. The self-expandable valves include the CoreValve, Evolut R, and Evolut PRO, while the balloon-expandable valves include the Edwards Sapien, Sapien XT, and Sapien 3. The Lotus valve and Lotus edge are classified as mechanically expandable valves. The new-generation device includes Sapien 3, Evolut R, Evolut PRO, Lotus, and Lotus Edge. The valves with leakage-proof function include Sapien 3, Evolut PRO, Lotus, and Lotus Edge. The primary outcome of the study was significant PVL (≥mild PVL) at 1-month follow-up echocardiography. The prosthetic valve function and PVL were evaluated by TEE and transthoracic echocardiography. The severity of PVL was assessed according to the Valve Academic Research Consortium-2 criteria [15]; none, no regurgitant flow; mild, the extent of regurgitation ˂10% of circumference; moderate, 10–29% of circumference; severe, ≥30% of the circumference. The OI was calculated as TAVI valve size (provided by the manufacturer)/MDCT annular size. The percentage of oversizing (positive OI) or undersizing (negative OI) was calculated differently according to the type of valve. The perimeter OI was calculated using the following formula in patients who received the CoreValve-Evolut system: ((nominal valve perimeter−measured perimeter)/nominal valve perimeter) × 100. The area OI in patients who received the Sapien and Lotus systems was calculated as follows: ((nominal valve area-measured area)/nominal valve area) × 100. The aortic annulus eccentricity index was calculated as 1 ‒(minimum diameter/maximum diameter) × 100. The calcium difference was defined as the calcium difference in calcium amount between the maximum and minimum calcium in each cusp. The annulus perimeter and area were measured in a short-axis plane during mid-systole.

### 2.3. Statistical Analysis

Data were expressed as numbers and percentages for categorical variables and as mean ± standard deviation or median and interquartile ranges (IQRs) for continuous variables. The differences in characteristics between groups were compared using chi-square tests for categorical variables and Student’s *t*-test for continuous variables. To determine the best cutoff value (COV) for OI to predict significant PVL (≥mild PVL), a receiver operating characteristic (ROC) curve analysis was performed. To identify the independent predictors of significant PVL (≥mild PVL), a multivariable binary logistic model was used. All variables clinically relevant to PVL and variables with a *p*-value of ≤0.1 in the univariate analysis were included in multivariable logistic regression analysis. The variables included valve without leakage-proof function (or self-expandable valve after sorting by valve with leakage-proof function), OI, total calcium amount, calcium difference, LVOT calcification, commissure calcification, and eccentricity index. The final models were determined using the forward stepwise method. Results were reported as odds ratios (ORs) and 95% confidence intervals (CIs). The association between OI and PVL was modeled with logistic regression, and non-linearity was explored using multivariable fractional polynomials. To prove the incremental value of OI in predicting PVL, category-free net reclassification index and integrated discrimination improvement analysis were performed. A two-sided value of *p*-value < 0.05 was considered significant for all probability values. The SPSS version 23 (IBM SPSS Statistics, Chicago, IL, USA) and STATA version 15 (StataCorp LLC, College Station, TX, USA) were used for all statistical analyses.

A comprehensive methods section can be found in the Supplementary Materials.

## 3. Results

### 3.1. Baseline Patient, Device, and Procedural Factors

**Figure 1:**
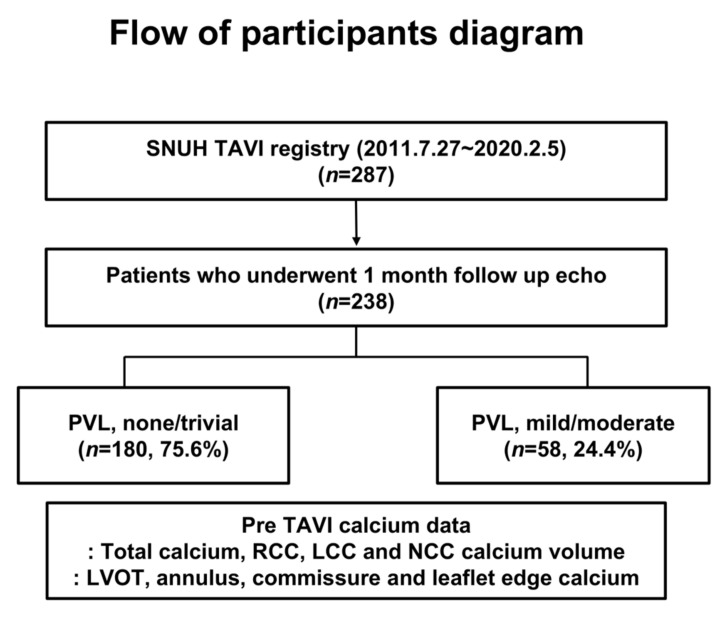
Flow diagram of participants. LCC, left coronary cusp; LVOT, left ventricular outflow tract; NCC, non-coronary cusp; PVL, paravalvular leakage; RCC, right coronary cusp; TAVI, transcatheter aortic valve implantation.

Patients who underwent 1-month follow-up echocardiography after TAVI were enrolled in this study (*n* = 238, Figure 1). The baseline characteristics according to presence or absence of significant PVL are shown in Table 1. The median age of the study population was 78.5 years, and 52.5% of patients were men. The valves included in the present study were Edwards Sapien (*n* = 11, 4.6%), Sapien XT (*n* = 4, 1.7%), Sapien 3 (*n* = 88, 37.0%), CoreValve (*n* = 37, 15.5%), Evolut R (*n* = 56, 23.5%), Evolut PRO (*n* = 19, 8.0%), Lotus (*n* = 20, 8.4%), and Lotus Edge (*n* = 3, 1.3%). Thus, 130 patients (54.6%) were implanted with a leakage-proof valve, while 52 (21.8%) were implanted with an early generation valve. The overall median OI was 13.5%.

At 1-month follow-up, 58.4% of the patients (*n* = 139) had no PVL, 17.2% (*n* = 41) had trace PVL, 19.4% (*n* = 46) had mild PVL, 4.2% (*n* = 10) had mild to moderate PVL, and 0.8% (*n* = 2) had moderate PVL. None of the patients had severe PVL. The patients were divided into two groups according to the grade of PVL: the group with significant PVL (≥mild PVL, 24.4%) and the group without PVL (none or trivial, 75.6%).

The baseline characteristics and echocardiographic variables were similar between the two groups. The median OI was smaller in the significant PVL than in the no PVL group (significant PVL group vs. no PVL group, 12.8% vs. 13.7%, *p* = 0.028). PVL was not associated with pre- and post-procedural mean pressure gradient or pre- and post-aortic regurgitation index. The significant PVL group showed a higher rate of self-expandable valve and early-generation valve implantation than the no PVL group. The valve with the leakage-proof function was less commonly used in the significant PVL than in the no PVL group (significant PVL vs. no PVL group, 39.7% vs. 59.4%, *p* = 0.008). The mean prosthetic valve size was similar between the two groups.

In the baseline computed tomography data, the median eccentricity index was 18.8 (IQR, 13.7–23.3), and the aortic annulus perimeter was 74.1 mm (IQR, 69.4–79.7) (Table 2). The median calcium volume within the area of interest was 535.3 mm^3^ (IQR, 305.8–905.6), and the volume of mean calcium per cusp was 181.1 mm^3^ (IQR, 103.5–313.8). The calcium difference among cusps was greater in the significant PVL group than in the no PVL group without statistical significance (*p* = 0.202). Overall, 89 patients (40.5%) showed commissural calcification, and it was more common in the significant PVL group than in the no PVL group (*p* = 0.019).

**Figure 2:**
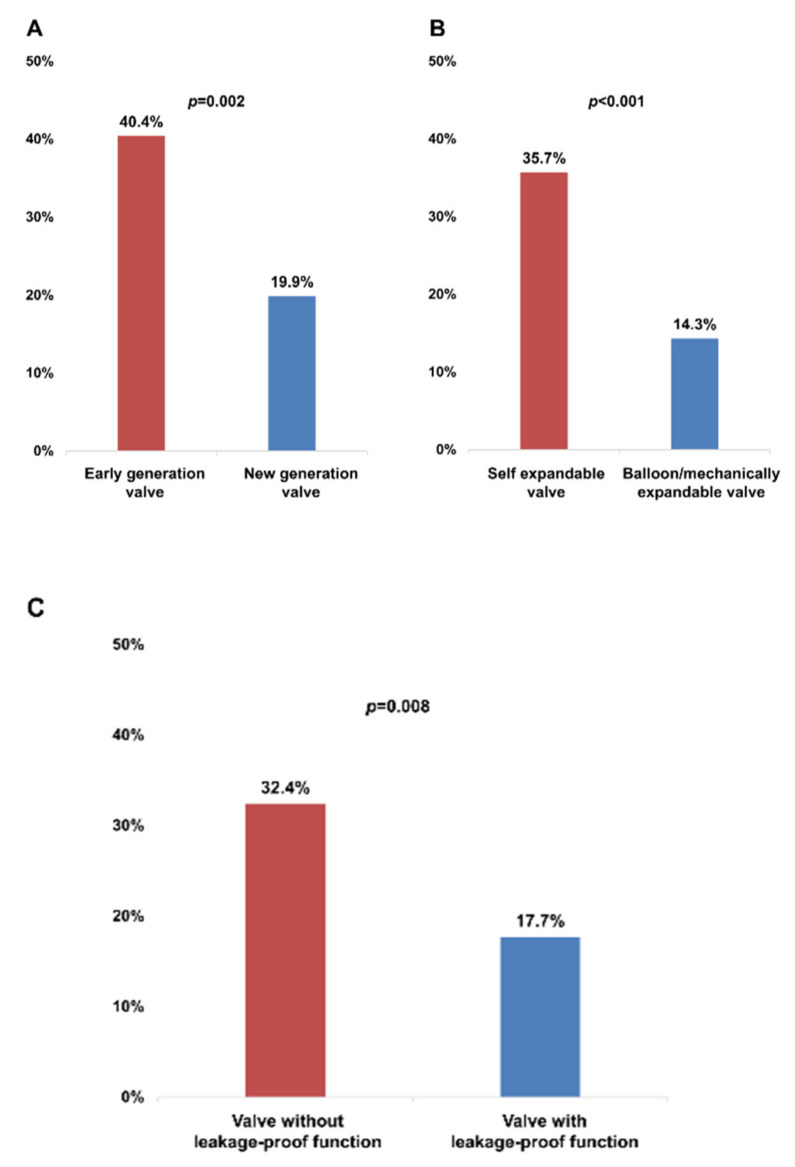

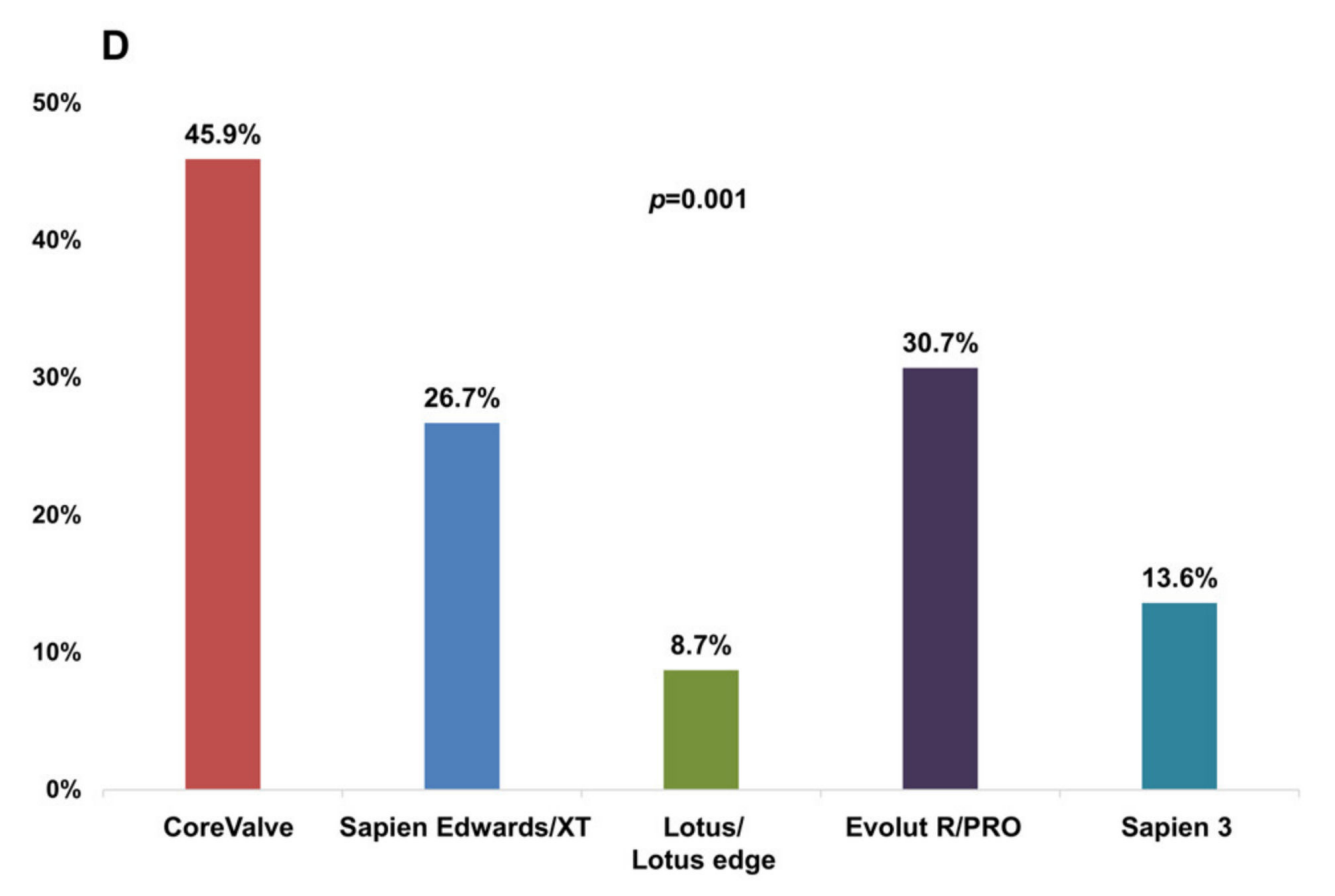
Incidence of significant PVL in each prosthesis group assessed by 1-month follow-up echocardiography. (**A**) early generation versus new-generation valve; (**B**) self-expandable versus balloon/mechanically expandable valve; (**C**) valve without leakage-proof function versus valve with leakage-proof function; (**D**) in the five prosthesis groups. PVL, paravalvular leakage.

Figure 2 shows the incidence of PVL depending on valve type. The incidence of significant PVL was higher in patients using the early generation valve than in those using the new-generation valve (40.4% vs. 19.9%, *p* = 0.002, Figure 2A), in patients using the self-expandable valve than in those using the balloon-expandable valve (35.7% vs. 14.3%, *p* ˂ 0.001, Figure 2B), and in patients using a valve without leakage-proof function than in those using a valve with leakage-proof function (32.4% vs. 17.7%, *p* = 0.008, Figure 2C). Among the three different brands of TAVI valve, the Lotus brand had a tendency with a lesser risk for PVL, whereas the Evolut brand was associated with a higher risk for PVL (Figure 2D). However, the incidence of permanent pacemaker (PPM) insertion within 1 month was comparable between the two different valve types using less OI in valves with leakage-proof function (valves with leakage-proof function vs. valves without leakage-proof function, OI, 12.1 ± 6.2% vs. 15.9 ± 7.9%, *p* < 0.001, the incidence of PPM insertion, 5.4% vs. 5.6%, *p* = 0.954).

### 3.2. Multivariable Model for the Prediction of Paravalvular Leakage

Table 3 summarizes the results of the multivariable model for the predictors of PVL. The possible predictors for PVL included in the multivariable model were factors from patients (total calcium amount, calcium difference between leaflets, commissure calcification, LVOT calcification, and eccentricity index of the annulus), device (valve with leakage-proof function or self-expandable valve), and procedure (OI). In the logistic regression model for the entire population, any patient factor was not a predictor of PVL. Meanwhile, the device or procedure factors were significant. The valve without leakage-proof function was associated with significant PVL (adjusted OR, 3.194, 95% CI, 1.620–6.299, *p* = 0.001). Lower OI was associated with significant PVL. The incidence of significant PVL increased by 45% when OI decreased by 5% (adjusted OR, 1.451, 95% CI, 1.154–1.825, *p* = 0.001). In valves without leakage-proof function, a total calcium amount of >800 mm^3^ (adjusted OR, 4.217, 95% CI, 1.519–11.708, *p* = 0.006) and a lower OI (OI per a decrease by 5%, adjusted OR, 1.645, 95% CI, 1.188–2.278, *p* = 0.003) were associated with significant PVL. However, in patients who received a valve with a leakage-proof function, the independent factors of significant PVL were self-expandable valve (Evolut PRO) and lower OI.

### 3.3. Optimal Oversizing Index for Different Valve Types to Prevent Paravalvular Leakage

**Figure 3:**
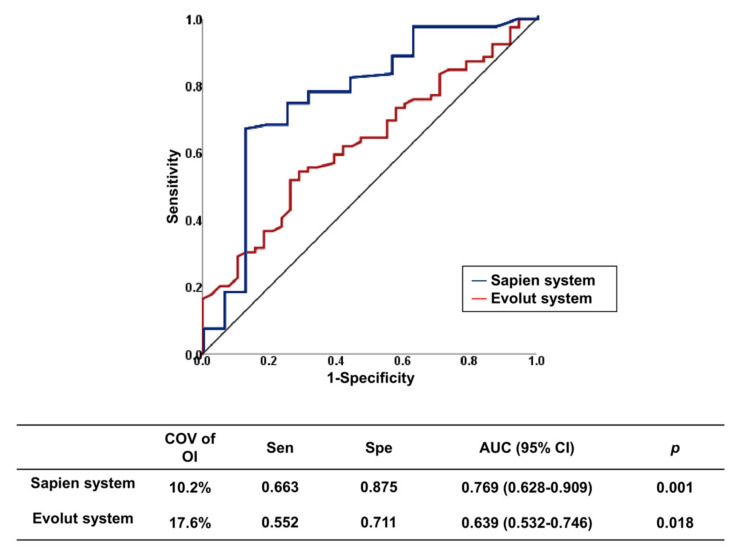
Receiver operating characteristic curves of optimal oversizing index for prediction absence of paravalvular leakage in each type of TAVI valve. AUC, area under the curve; CI, confidence interval; COV, cut-off value; TAVI, transcatheter aortic valve implantation.

**Figure 4:**
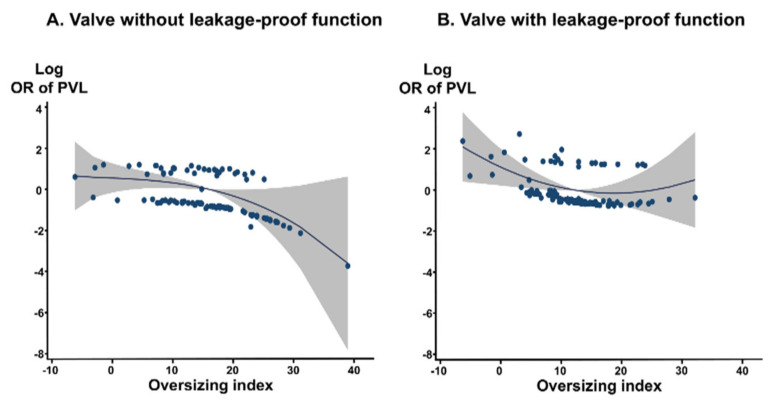
Continuous relation between oversizing index and risk of PVL calculated by fractional polynomial regression. Gray shading indicates 95% confidence interval around the flat line. The OR was adjusted for the calcium amount. (**A**) valve with leakage-proof function; (**B**) valve with leakage-proof function. OR, odds ratio; PVL, paravalvular leakage.

Device type and OI are predictors of PVL. We analyzed the optimal OI predicting the absence of PVL for each valve type. These COVs are summarized in Table 4. For valve without leakage-proof function, the COV of OI for predicting no PVL was 17.3%, with an area under the ROC curve (AUC) of 0.679 (*p* = 0.004). For the valve with leakage-proof function, there was no appropriate COV of OI, suggesting that sizing may be less important for PVL in these valves with leakage-proof function. The COVs for predicting no PVL differed between self-expandable versus balloon-expandable valves. The COVs of OI for predicting no PVL were ≥17.6% in the Evolut system and ≥10.2% in the Sapien system (AUC, 0.639, *p* = 0.018 in the Evolut system, AUC, 0.769, *p* = 0.001 in the Sapien system) (Figure 3). For the Sapien 3 system, the optimal COV of OI was ≥10.2%, which is similar to that of the Sapien system (AUC, 0.755, *p* = 0.005 in Sapien 3). The incremental values of OI over the reference variable were observed for the entire population and for each type of valve with or without leakage-proof function (Table 5). PVL decreased when the OI increased up to 30% in valves without leakage-proof function (Figure 4A). In valves with leakage-proof function, the PVL decreased when the OI increased to 10%, and then showed no further decrease even though the OI increased further to 30% (Figure 4B).

### 3.4. Association of PVL Predictors and in-Hospital PPM Implantation

In-hospital PPM implantation was performed in 5.0% (*n* = 12) of patients after TAVI. However, there was no significant difference in OI depending on PPM insertion (PPM insertion vs. no insertion, OI, 14.2 ± 10.8% vs. 13.7 ± 7.0%, *p* = 0.893). There was no difference in the volume of calcium (PPM insertion vs. no insertion, 351 mm^3^ vs. 551 mm^3^, *p* = 0.776) and presence of LVOT calcification (PPM insertion vs. no insertion, 8.3% vs. 13.9%, *p* = 1.000) depending on PPM insertion.

## 4. Discussion

The important points of this study were as follows: (1) TAVI valve with leakage-proof function showed lower PVL incidence than without leakage-proof function; (2) Considering PVL, the appropriate increase in OI may result in lower PVL incidence. The best COV of OI for predicting no/trace PVL was ≥17.3% for valves without leakage-proof function. The best COV of OI to predict the absence of PVL were ≥17.6% for Evolut system and ≥10.2% for Sapien system; (3) The important risk factor for PVL in valves without leakage-proof function was the large amount of calcium on top of insufficient OI, while that in valve with the leakage-proof function was a self-expandable type of valve on top of insufficient OI; (4) The upper limit of OI to prevent PVL was 30% or higher in valves without leakage-proof function, while it was around 15% in valves with leakage-proof function. The present study demonstrates that appropriate selection of device (valve with leakage-proof function) and procedural (OI) factors is important to prevent PVL after TAVI.

Although the indication for TAVI has evolved as a new standard for the treatment of severe AS patients, PVL is considered the Achilles’ heel of TAVI. Surgical AV replacement could achieve less PVL than TAVI due to resection of the calcified AV leaflet, annulus debridement, and suturing to obliterate the gap around the prosthesis [16]. Regular sutures minimize the PVL by fixing the new AV to decalcified annulus [16]. PVL is an important complication of TAVI because it is associated with higher mortality during follow-up. Similar to moderate to severe PVL, mild or greater PVL is also associated with mortality after TAVI [1,6,7]. Based on the data from the PARTNER trial, the PVL group had worse 1-year mortality and re-hospitalization rate than the no PVL group [6]. A novel role for von Willebrand Factor (vWF), the mediator of hemostasis and vascular inflammation, is emerging as a useful sensor in the assessment of PVL after TAVI [17,18]. In patients who have received a successful TAVI, HMWM (high-molecular-weight multimers) -vWF was normalized within a few minutes after the TAVI procedure. If PVL is still present after the TAVI procedure, defect of HMWM-vWF persists. vWF could be real-time detector for PVL. The association between PVL and mortality remains unclear, but some explanations exist. In an experimental model, PVL after TAVI results in poor coronary perfusion during the diastolic phase and greater preload on LV, which consequently imposes a higher workload on the LV [8]. Patients with PVL after TAVI showed greater LV end-diastolic dimension and less regression of LV mass than those without PVL [8]. The factors associated with PVL were derived from patient, device, and procedure. Patient factors cannot be modified, and attention should be paid to the device or procedural factors that can be modifiable to reduce PVL. In our analysis, these factors were optimal OI based on accurate aortic annulus measurement and selection of valves with leakage-proof function.

Patient factors related to PVL included gender, AV leaflet calcification, LVOT eccentricity, and discordant sizing between the annulus and LVOT oversizing. Women generally have higher perioperative morbidity and mortality after AV replacement surgery, but the clinical outcomes of females after TAVI are better than males, except for vascular complications [19]. In women, smaller aortic annulus and sinus of Valsalva are associated with a higher oversizing index and may lower the risk of PVL [20]. Consistent with the previous study, our results also showed that women have a larger mean OI than men’s (women vs. men, 16.4 ± 8.5% vs. 12.4 ± 7.2%, *p* ˂ 0.001). Some studies have shown that a large volume of AV calcium is associated with PVL [13,21], suggesting that the location of the PVL was associated with the cusp with the highest calcium amount. However, in our previous study, the eccentric distribution of calcium based on the bi-partition method was an important predictor for PVL [22]. Our results also showed that a large calcium volume is also a predictor of PVL in valves without leakage-proof function. However, the amount of calcium does not matter in valves with leakage-proof function. Furthermore, calcification has different effects on PVL depending on the calcium location. Valvular calcification is not associated with PVL because the calcified valve is displaced to the sinus of Valsalva if the anchoring and sealing were perfect within the annulus or LVOT [23]. Several studies examined the importance of LVOT calcium [21,23,24]. A previous study on device landing zone calcification and PVL showed that AV calcification is related to PVL only in the presence of LVOT calcification (COV > 10 mm^3^) [24]. In this study, the procedures were almost performed using valves without leakage-proof function. Although our study did not show an association between total calcium volume and PVL, we found that the commissural calcification was associated with PVL. However, this factor disappeared when we performed the multivariable analysis adjusted for covariates including valves with or without leakage-proof function and OI. In a recent study with Edwards SAPIEN 3 valve, three-leaflet calcification, LVOT eccentricity, and annulus-LVOT size mismatching were independent predictors of 1-month PVL [25]. This study showed that not only annulus size or calcification, but also LVOT sizing is important for preventing PVL.

Because PVL, after the TAVI procedure, was associated with adverse outcomes, several next-generation devices were developed to reduce the incidence of PVL using new methods of anchoring or sealing, repositioning, and inflatable cuffs [26]. Sapien 3 with an additional outer skirt, a polyethylene terephthalate, has a lesser incidence of moderate to severe PVL than Sapien XT especially in patients with significant AV calcification [13,27]. Evolut PRO has an external wrap that increases surface contact and reduces the gap between native annulus and prosthesis, leading to a reduction of PVL [28]. Lotus valve incorporates a polymeric outer adaptive seal designed to lower PVL [29]. In this study, the incidence of PVL at 1 month was significantly lower in valves with leakage-proof function than in those without leakage-proof, which was more dramatic in the Sapien/Lotus system than in the Evolut system.

Moderate oversizing of the TAVI valve relative to the aortic annulus dimensions is generally accepted in order to reduce the PVL [12,13]. However, optimal OI has not been studied or proposed. In a previous study using the Sapien XT valve, patients with moderate to severe PVL had lower OI than those with less PVL [12]. The appropriate oversizing was at least 1 mm over the mean annular diameter and at least 10% over the mean annular area [12]. In this study, we attempted to determine the optimal OI to reduce PVL in different types of TAVI valves. In the multivariate analysis, the OI and valves with leakage-proof function were the two determinants of significant PVL, while calcium distribution and amount were not significant. This finding may suggest that patients’ anatomic factors can be overcome by appropriate oversizing of the contemporary TAVI device. Then, we defined an appropriate OI for the first time. In order to prevent PVL, the OI should be at least 17.3% in the early generation valve without leakage-proof function, whereas no appropriate COV was established in valves with leakage-proof function, suggesting that the influence of OI may be weakened due to the presence of leakage-proof function. Thus, in our data, a lower mean OI was applied to valves with leakage-proof function than in those without leakage-proof. However, the incidence of significant PVL at 1 month was actually lower in the valve with leakage-proof function. Our fractional polynomial regression graphs proved that the larger OI, the better in the case of valves without leakage-proof function. However in valves with leakage-proof function, proper OI (10–15%) is sufficient to achieve good results. Newer-generation valves with leakage-proof functions provide sufficient anchoring and obliteration of gap between tissue and prosthesis, require less OI, and result in lower PVL rates without increasing PPM insertion rates. Furthermore, the optimal COVs of OI were 10.2% in the Sapien system including Sapien 3 and 17.6% in the Evolut system.

However, excessive OI may not good for the TAVI procedure. In a previous study using a cardiovascular simulator, implantation of a big transcatheter heart valve into a small annulus may induce a mismatch between the leaflet and diameter of valve frame, plication of the leaflets, and under-expansion of valve frame, leading to poor hemodynamics [30]. Moreover, it is important to perform MDCT prior to TAVI because the aortic annulus has an oval shape structure, and the annulus size measured by echocardiography is smaller than the annulus diameter of MDCT [12,31].

This study had some limitations. First, the favorable outcome of the leakage-proof valve may be influenced by the accumulating procedure experiences including patient selection and pre-procedural imaging modalities. Second, valve selection was not random, which may have resulted in selection bias. Third, this study was conducted with relatively small numbers of study populations, and a particularly small number of patients using Evolut PRO and Lotus Edge were included in this study. However, as far as we know, this is one of the few studies to study the effects of OI in patients who underwent TAVI procedures. Fourth, although moderate or higher PVL is known to be associated with a poor prognosis, the long-term effect of mild PVL is still not widely known. Lastly, we did not know the maximal upper limit of OI due to the risk of annulus rupture, which warrants further bench tests using phantom models and computed hemodynamics.

## 5. Conclusions

The predictors of PVL after TAVI were more associated with device factors (without leakage-proof function) and procedural factors (under-sizing) rather than patient factors (anatomic factors). Appropriate procedures using contemporary devices may overcome the patient’s anatomic factors such as commissure calcification. Therefore, for patients at high risk of PVL, the procedure should be optimized using valves with leakage-proof function and adequate OI.

## Figures and Tables

**Table 1:** Baseline Clinical and Procedural Characteristics.

	Total (*n* = 238)	PVL Mild/Moderate (*n* = 58)	PVL None/Trivial (*n* = 180)	*p*
Age (years)	78.5 (74.0–83.0)	78.0 (71.8–82.3)	79.0 (74.0–83.0)	0.513
Male	125 (52.5)	28 (48.3)	97 (53.9)	0.457
Diabetes mellitus	80 (33.6)	21 (36.2)	59 (32.8)	0.631
Hypertension	171 (72.5)	48 (82.8)	123 (69.1)	0.043
Previous PCI	71 (30.1)	14 (24.1)	57 (32)	0.256
Previous CABG	6 (2.5)	0 (0)	6 (3.3)	0.340
Valve in valve	8 (3.4)	2 (3.4)	6 (3.3)	1.000
Previous AVR	7 (2.9)	1 (1.7)	6 (3.3)	1.000
Previous TAVI	1 (0.4)	1 (1.7)	0 (0)	0.244
Previous heart surgery	13 (5.5)	2 (3.4)	11 (6.1)	0.740
Previous hemodialysis	14 (6.0)	5 (8.9)	9 (5.0)	0.331
Atrial fibrillation	24 (10.1)	8 (13.8)	16 (8.9)	0.281
Previous PPM	6 (2.5)	0 (0)	6 (3.4)	0.340
Tricuspid aortic valve	199 (83.6)	52 (89.7)	147 (81.7)	0.153
EuroSCORE II (%)	3.0 (1.8–5.5)	3.1 (1.9–4.7)	2.9 (1.8–6.0)	0.298
STS PROM score (%)	3.9 (2.5–8.1)	3.9 (2.2–9.7)	3.9 (2.5–7.8)	0.633
Echocardiographic variables				
Peak AV velocity (m/s)	4.4 (4.1–5.0)	4.5 (4.1–5.2)	4.4 (4.1–5.0)	0.566
Mean gradient (mmHg)	48.0 (40.6–62.0)	48.3 (39.4–62.8)	48.0 (40.8–61.5)	0.574
LV ejection fraction (%)	60 (55–65)	61 (55–65)	60 (55–65)	0.411
AV area (cm^2^)	0.7 (0.6–0.8)	0.7 (0.6–0.9)	0.7 (0.6–0.8)	0.606
Access site				0.046
Trans-femoral	234 (98.3)	55 (94.8)	179 (99.4)	
Trans-apical	4 (1.7)	3 (5.2)	1 (0.6)	
Procedural duration				
Puncture to close (mins)	63 (50–83)	68 (54–100)	60 (48–82)	0.011
Anesthesia time (mins)	131 (110–160)	140 (115–170)	130 (110–150)	0.041
Admission duration (days)	10 (9–14)	11 (9–15)	10 (9–14)	0.471
TAVI to discharge (days)	8 (7–10)	9 (7–12)	8 (7–10)	0.377
Pre-ballooning	87 (36.6)	27 (46.6)	60 (33.3)	0.069
Post-ballooning	89 (37.6)	27 (46.6)	62 (34.6)	0.103
Oversizing index (%)	13.5 (9.3–18.7)	12.8 (7.8–17.7)	13.7 (9.8–18.8)	0.028
Pre mean PG (mmHg)	50 (37–65)	49 (36–65)	50 (37–64)	0.442
Post mean PG (mmHg)	5 (2–9)	7 (1–11)	5 (2–8)	0.184
delta mean PG (mmHg)	44 (29–59)	42 (26–62)	44 (30–59)	0.864
Pre AR index	26 (19–32)	25 (18–33)	26 (19–32)	0.955
Post AR index	28 (23–33)	27 (23–33)	28 (23–33)	0.747
delta AR index	2 (−2–8)	3 (−4–6)	2 (−2–9)	0.612
TAVI valve depth				
NCC depth (mm)	3.8 (2.8–5.5)	4.0 (2.5–6.4)	3.8 (2.9–5.2)	0.255
RCC depth (mm)	3.7 (2.8–5.0)	3.9 (2.7–7.4)	3.7 (2.8–5.0)	0.266
Leakage proof valve	130 (54.6)	23 (39.7)	107 (59.4)	0.008
Early generation valve	52 (21.8)	21 (36.2)	31 (17.2)	0.002
Self-expandable valve	112 (47.1)	40 (69.0)	72 (40.0)	<0.001
Lotus system	23 (9.7)	2 (3.4)	21 (11.7)	0.065
Mean valve size (mm)	26.3 ± 2.4	26.7 ± 2.7	26.1 ± 2.3	0.157

Values are mean ± SD, median (interquartile ranges, 25–75th), or *n* (%). AR, aortic regurgitation; AV, aortic valve; AVR, aortic valve replacement; CABG, coronary artery bypass surgery; LV, left ventricle; LVOT, left ventricular outflow tract; NCC, non-coronary cusp; PCI, percutaneous coronary intervention; PG, pressure gradient; PPM, permanent pacemaker; PVL, paravalvular leakage; RCC, right coronary cusp; STS PROM, Society of Thoracic Surgeons Predicted Risk of Mortality models; TAVI, transcatheter aortic valve implantation.

**Table 2:** Baseline computed tomography. Characteristics according to presence or absence of PVL.

	Total (*n* = 238)	PVL Mild/Moderate (*n* = 58)	PVL None/Trivial (*n* = 180)	*p*
annulus min diameter, mm	21.2 (19.8–22.7)	21.3 (19.8–22.9)	21.2 (19.7–22.6)	0.540
annulus max diameter, mm	26.1 (24.3–28.2)	26.2 (24.7–28.5)	26.0 (24.2–28.1)	0.355
Eccentricity index, %	18.8 (13.7–23.3)	19.6 (13.0–23.7)	18.7 (14.0–23.3)	0.651
annulus perimeter, mm	74.1 (69.4–79.7)	74.4 (70.7–81.1)	73.7 (69.0–79.0)	0.163
LCA height, mm	12.3 (10.6–14.3)	12.8 (11.3–15.0)	12.1 (10.5–14.0)	0.428
RCA height, mm	15.0 (13.0–17.5)	15.3 (12.6–17.7)	15.0 (13.0–17.4)	0.990
STJ diameter, mm	27.1 (24.4–29.5)	27.6 (24.9–30.9)	26.9 (24.4–29.1)	0.087
SV diameter, mm	32.9 (30.3–35.6)	34.3 (30.7–36.7)	32.6 (30.0–35.5)	0.490
Calcium data				
Total calcium, mm^3^	535.3 (305.8–905.6)	559.1 (287.5–1011.3)	531.8 (310.9–849.2)	0.468
LCC calcium, mm^3^	124.8 (60.7–271.1)	120.6 (42.0–333.5)	127.2 (64.2–262.0)	0.325
RCC calcium, mm^3^	146.5 (69.9–303.5)	153.3 (72.5–336.3)	144.6 (65.6–275.2)	0.428
NCC calcium, mm^3^	214.8 (117.9–382.8)	213.6 (104.8–372.9)	216.8 (120.3–385.9)	0.976
Mean calcium/cusp, mm^3^	181.1 (103.5–313.8)	193.8 (98.5–337.1)	180.8 (104.1–311.4)	0.366
Ca difference, mm^3^	170.6 (85.4–293.2)	188.3 (80.4–342.3)	155.3 (88.6–282.6)	0.202
LVOT calcification	30 (13.6)	11 (20.4)	19 (11.4)	0.097
Annulus calcification	48 (21.8)	16 (29.6)	32 (19.3)	0.110
Commissure calcification	89 (40.5)	28 (51.9)	61 (36.7)	0.019
Leaflet edge calcification	142 (82.1)	34 (79.1)	108 (83.1)	0.553

LCA, left coronary artery; LCC, left-coronary cusp; LVOT, left ventricular outflow tract; NCC, non-coronary cusp; RCA, right coronary artery; RCC, right coronary cusp; STJ, sinotubular junction; SV, sinus of Valsalva.

**Table 3:** Independent factors for mild/moderate para-valvular leakage at logistic regression analysis.

	Univariable Analysis		Multivariable Analysis	
	OR (95% CI)	*p*	OR (95% CI)	*p*
Total population				
Valve without leakage proof function	2.237 (1.211–4.130)	0.010	3.194 (1.620–6.299)	0.001
OI per a decrease by 5%	1.306 (1.053–1.618)	0.015	1.451 (1.154–1.825)	0.001
Total calcium amount >800 mm^3^	1.392 (0.718–2.697)	0.328		
Calcium difference >200 mm^3^	1.231 (0.666–2.277)	0.507		
LVOT calcification	1.979 (0.875–4.479)	0.101		
Commissure calcification	1.854 (0.997–3.446)	0.051		
Eccentricity index	1.003 (0.960–1.048)	0.889		
Valve without leakage-proof function				
Total calcium amount >800 mm^3^	3.684 (1.430–9.492)	0.007	4.217 (1.519–11.708)	0.006
OI per a decrease by 5%	1.578 (1.165–2.138)	0.003	1.645 (1.188–2.278)	0.003
Calcium difference >200 mm^3^	2.088 (0.894–4.879)	0.089		
Commissure calcification	1.538 (0.658–3.595)	0.320		
LVOT calcification	1.508 (0.516–4.404)	0.453		
Self-expandable valve	1.399 (0.410–4.780)	0.592		
Eccentricity index	0.998 (0.948–1.051)	0.946		
Valve with leakage-proof function				
Evolut PRO vs. Lotus/Sapien 3	6.171 (2.136–17.828)	˂0.001	27.670 (5.536–138.311)	˂0.001
OI per a decrease by 5%	1.270 (0.886–1.821)	0.193	2.983 (1.537–5.790)	0.001
LVOT calcification	2.379 (0.657–8.613)	0.187		
Commissure calcification	1.582 (0.592–4.229)	0.360		
Calcium difference >200 mm^3^	0.540 (0.195–1.496)	0.236		
Total calcium amount >800 mm^3^	0.472 (0.148–1.509)	0.206		
Eccentricity index	0.997 (0.923–1.078)	0.950		

CI, confidence interval; LVOT, left ventricular outflow tract; OI, oversizing index; OR, odds ratio.

**Table 4:** The cut-off value of oversizing index of predicting absence of paravalvular leakage.

	COV	Sen	Spe	AUC	95% CI	*p*
Valve without leakage-proof function	17.3	0.544	0.727	0.679	0.570–0.788	0.004
Valve with leakage-proof function	-	-	-	0.558	0.410–0.707	0.381
Sapien system	10.2	0.663	0.875	0.769	0.628–0.909	0.001
Sapien 3	10.2	0.645	0.917	0.755	0.602–0.908	0.005
Evolut system (including CoreValve)	17.6	0.552	0.711	0.639	0.532–0.746	0.018
Evolut R/PRO	-	-	-	0.601	0.462–0.741	0.184

AUC, area under the curve; CI, confidence interval; COV, cut-off valve; Sen, sensitivity; Spe, specificity.

**Table 5:** Incremental value of oversizing index over reference value for predicting no PVL.

Parameters	AUC		Category-Free NRI		IDI	
	Value	*p*	Value	*p*	Value	*p*
Total population						
Valve with leakage proof function *	0.599	0.009				
Reference + oversizing index	0.676	<0.001	0.340	0.027	0.052	0.002
Valve without leakage proof function						
Total calcium amount ≤800 *	0.631	0.005				
Reference + oversizing index	0.752	<0.001	0.387	0.068	0.093	0.004
Valve with leakage proof function						
Non Self-expandable valve *	0.648	0.001				
Reference + oversizing index	0.806	<0.001	0.769	<0.001	0.098	0.010

* Results of marked variable were taken as reference values for category-free NRI and IDI analyses. IDI, integrated discrimination improvement; NRI, net reclassification index; PVL, paravalvular leakage.

## Supplementary Materials

The following are available online at https://www.mdpi.com/2077-0383/9/12/3936/s1, eMethods.
