# Supplementary material for: Optimal Oversizing Index Depending on Valve Type and Leakage-Proof Function for Preventing Paravalvular Leakage after Transcatheter Aortic Valve Implantation
Source: J Clin Med. 2020 Dec 4;9(12):3936. doi: 10.3390/jcm9123936 (PMC7761987; doi:10.3390/jcm9123936)
Supplement: Supplementary file 1 [file jcm-09-03936-s001.pdf]

<u>Supplement to</u>:

Optimal oversizing index depending on valve type and leakage-proof function for preventing paravalvular leakage after transcatheter aortic valve implantation

You-Jeong Ki, MD, Jeehoon Kang, MD, Hak Seung Lee, MD, Mineok Chang, MD, PhD, Jung-Kyu Han, MD, PhD, Han-Mo Yang, MD, PhD,

Kyung Woo Park, MD, PhD, Hyun-Jae Kang, MD, PhD, Bon-Kwon Koo, MD, PhD, Hyo-Soo Kim, MD, PhD

<u>Contents</u>:

Page 2-4: eMethods

eMethods

Devices, procedures, and data collection

Computed tomography (CT) imaging was used to assess the anatomical feasibility of TAVI (transcatheter aortic valve implantation) and determine the access site and sheath type. The device and size were selected based on the CT and transesophageal echocardiography (TEE) findings. The baseline, procedural, and CT characteristics, and follow-up data were prospectively collected in a standardized database. Depending on the patients' or anesthesiologists' preference, general and local anesthesia were used under monitored anesthesia care. TEE was used to assess the correct valve size, valve position, and valvular function. Pre-balloon dilatation prior to valve implantation was performed according to the operators' decision. After valve implantation, the prosthetic valve position and function were confirmed using fluoroscopy and TEE. If there was an incomplete expansion of the valve or more than mild paravalvular leak (PVL), post-dilatation with a balloon was performed. Clinical follow-up data were obtained via outpatient visits, telephone calls, and/or medical questionnaires.

Computed tomography angiography

Pre-procedural CT angiography imaging was performed using a 64-channel dual-source CT scanner (SOMATOM Definition, Siemens Medical Solutions, Forchheim, Germany; or Aquilion ONE, Toshiba Medical System, Japan). The scanning protocols for CT were as follows:

craniocaudal direction with retrospective electrocardiogram-gating, bolus tracking in the descending aorta with a trigger value of 100–150 Hounsfield units (HU), tube voltages of 100–120 kVp, automatic tube current for patient size, slice thicknesses of 0.6 mm for SOMATOM Definition and 1.0 mm for Aquilion ONE, 0.2 pitch, gantry rotation time of 280 or 350 ms, and reconstruction interval of 0.6 mm or 1.0 mm, for covering subclavian artery to heart.

Calcium quantification

We quantified the aortic valve (AV) calcium mass in cubic millimeters (mm^3) using 3mensio Structural Heart software (version 7.0 SPI, Pie Medical Imaging BV, Maastricht, The Netherlands). After designation of the basal plane, the lower coronary ostium was determined, and the area of interest (AOI) was set before the origin of the coronary vessel. The aortic annulus was defined as a virtual plane containing low attachment points of the three aortic valves. The 3mensio software can automatically delineate the AV cusp and determine the volume of calcium using the set in HU at each AV cusp. The AOI consisted of the left ventricular outflow tract (LVOT) (3 mm inferior from the basal plane), aortic annulus, valvular cusps, and commissure. The calcium distribution within the AOI was divided into left coronary cusp, right coronary cusp, and non-coronary cusp. The calcium amount in each of the cusps was calculated. The LVOT, annulus, commissure, and leaflet edge calcification were also qualitatively confirmed. Every voxel above the threshold of 850 HU was considered a calcification. Except for patients who had previously

had a valve implantation and had difficulty measuring the calcium amount, a threshold of 850 HU was used for 129 patients (58.4%). In the remaining 92 patients (41.6%), we adjusted the threshold to better reflect the distribution of calcification. All voxels exceeding the threshold HU were summed to calculate the total AV calcification.
